# Strategies to enhance THz harmonic generation combining multilayered, gated, and metamaterial-based architectures

**DOI:** 10.1038/s41377-024-01657-1

**Published:** 2025-01-09

**Authors:** Ali Maleki, Moritz B. Heindl, Yongbao Xin, Robert W. Boyd, Georg Herink, Jean-Michel Ménard

**Affiliations:** 1 Department of Physics, University of Ottawa, Ottawa, ON K1N 6N5 Canada; 2 Experimental Physics VIII – Ultrafast Dynamics, University of Bayreuth, Bayreuth, 95447 Germany; 3 Iridian Spectral Technologies Ltd, Ottawa, ON K1G 6R8 Canada; 4 School of Electrical Engineering and Computer Science, University of Ottawa, Ottawa, ON K1N 6N5 Canada; 5 Institute of Optics and Department of Physics and Astronomy, University of Rochester, Rochester, NY 14627 USA

**Keywords:** Optical properties and devices, Nonlinear optics, Terahertz optics, Photonic devices, Metamaterials

## Abstract

Graphene has unique properties paving the way for groundbreaking future applications. Its large optical nonlinearity and ease of integration in devices notably makes it an ideal candidate to become a key component for all-optical switching and frequency conversion applications. In the terahertz (THz) region, various approaches have been independently demonstrated to optimize the nonlinear effects in graphene, addressing a critical limitation arising from the atomically thin interaction length. Here, we demonstrate sample architectures that combine strategies to enhance THz nonlinearities in graphene-based structures. We achieve this by increasing the interaction length through a multilayered design, controlling carrier density with an electrical gate, and modulating the THz field spatial distribution with a metallic metasurface substrate. Our study specifically investigates third harmonic generation (THG) using a table-top high-field THz source. We measure THG enhancement factors exceeding thirty and propose architectures capable of achieving a two-order-of-magnitude increase. These findings underscore the potential of engineered graphene-based structures in advancing THz frequency conversion technologies for signal processing and wireless communication applications.

## Abstract (web summary)

THz third harmonic generation setup. A lowpass filter transmits a multicycle THz pulse, a graphene-based sample efficiently enables third harmonic generation, and a highpass filter helps improve its detection.

## Graphical abstract

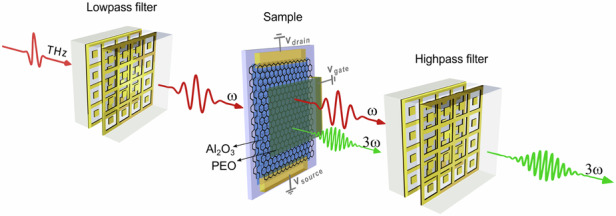

## Introduction

Nonlinear optics in the terahertz (THz) region has emerged as a promising field with diverse scientific and technological applications, fostering innovations in optical devices, advancements in material analysis, and imaging^1–3^. Intense THz fields interacting with nonlinear materials offer pathways for fundamental insights, from studying solid-state materials to capturing carrier and phonon dynamics^4–6^. To enable ultrahigh-speed information and communication technologies, many efforts are also invested to achieve efficient nonlinear THz frequency converters^7^. An archetypal illustration of this process is high harmonic generation (HHG), where resulting photons are generated at energies corresponding to multiples of the incident photons’ energy^8^.

Recent years have witnessed a broadening landscape of efficient THz HHG platforms relying on various materials. They include superconductors^9,10^, transition metal oxides (such as CaRuO_3_)^11^, bulk semiconductors^12^, and most notably, Dirac fermion systems. Dirac electronic band structures exhibit a linear energy-momentum dispersion that has been associated with a strong nonlinear response at THz frequencies. These effects have been studied in different material geometries, such as one-dimensional (1D) massless Dirac systems (e.g. carbon nanotubes)^13^, 2D semimetals and topological insulators (e.g. Bi_2_Se_3_)^14–16^, and 3D Dirac semimetals (e.g. Cd_3_As_2_)^17^. HHG in 2D Dirac-fermion graphene^18^ has been reported across a wide spectral range, from the near-infrared^19^, to the mid-infrared^20–22^ and the THz^23–25^ regions. One contribution to the optical nonlinearity of graphene can be closely linked to the transport dynamics of its carriers in response to an applied electric field. Unlike visible or NIR excitations leading to interband transitions, a THz driving field causes intraband transitions. As free carriers absorb energy through Drude absorption, this energy is redistributed, increasing the free-carrier temperature (exceeding 1000 K at THz field strength of 20 kV/cm)^26^ due to graphene’s low electronic heat capacity. This rise in temperature reduces THz conductivity and absorption, leading to a heating-cooling cycle that creates strong thermodynamic nonlinearity and resulting in harmonic generation at odd multiples of the THz driving field frequency^23,24,27^.

In the THz region, previous work on graphene relying on comparably narrowband accelerator-based super-radiant THz sources reported a strong nonlinear response (${\chi }^{\left(3\right)} \sim {10}^{-9}\,{\rm{m}}^{2}{\rm{/V}}^{2}$), resulting in an efficient THG conversion efficiency of 0.1%^24^. Remarkably, this nonlinear response in graphene is achievable at moderate THz pump field strengths ranging between 10 to 90 kV/cm, at room temperature, and under ambient conditions. To enhance nonlinear effects in graphene-based samples in the THz region, several approaches have been used. For example, previous work has shown that an optimization of the carrier density in graphene can enhance the power conversion efficiency of the THz THG^28^. Metasurfaces can also be used to locally enhance an incident pump field to enable stronger HHG in a graphene sheet, reaching up to a 1% field conversion efficiency at an incident field strengths <30 kV/cm^29^. While these approaches are efficient in boosting nonlinear effects, they have certain limitations. Notably, they have been demonstrated independently in the context of a single graphene layer. A multiple-layer graphene device could offer longer interaction lengths to improve frequency conversion efficiency. However, it remains unclear how such a design could be combined with other techniques to enhance nonlinear effects. Moreover, most studies reported on THz HHG have relied on accelerator-based super-radiant sources, which provide narrow-bandwidth (20% of the central frequency, FWHM) multicycle THz pulses with peak field amplitudes reaching up to 85 kV/cm. Here, we access the regime of THz HHG using a table-top configuration generating a 32 kV/cm peak field, which consists of an ultrafast near-infrared source, a high-field THz setup, and a combination of THz spectral filters. Most importantly, we experimentally explore how to efficiently combine different strategies to enhance these nonlinear effects.

Here, we investigate the enhancement of third harmonic generation (THG) in chemical vapor deposition (CVD) graphene through three distinctive approaches. We first investigate the nonlinear response of stacked decoupled graphene sheets, ranging from 1 to 15 layers. Results show a correlation between the field strength of the generated third harmonic and the increasing number of graphene layers with a maximum nonlinear signal observed for 6 layers, partially due to a trade-off between enhanced interaction length and linear absorption. Then, we use an electrical gate to investigate the effect of doping concentration in a 1-, 2-, and 3-layer graphene stack. Finally, we look at the effect of a metallic patterned substrate to locally enhance the THz driving field. We compare three types of metasurfaces acting on the THz driving field as a bandpass filter, a bandstop filter, and a linear wire-grid polarizer (WGP). The two kinds of spectral filters provide larger field enhancements than any previously reported metasurface substrates up to now used in these THz HHG experiments. A simple model is proposed to estimate the THG enhancement factor based on the metasurface geometry. This study on a various set of graphene-based architectures provides privileged insight on the combination of distinct experimental strategies to increase frequency conversion processes in 2D materials.

## Results

### Stacked graphene layers

We explore THG in CVD graphene within the THz region using a table-top time-resolved THz setup generating single-cycle pulses from a nonlinear lithium niobate crystal^30^ with a peak field of 360 kV/cm (see Methods). Multilayered graphene samples are fabricated on a THz transparent Zeonor substrate by successively depositing single graphene layers onto each other with the wet-transfer method (see Methods) while a 60 nm-thick aluminum oxide (Al_2_O_3_) spacing layers, only located between graphene sheets, reduces interlayer interactions. The total sample thickness remains significantly sub-wavelength, eliminating the need to satisfy phase-matching conditions to achieve coherent nonlinear effects.

**Fig. 1:**
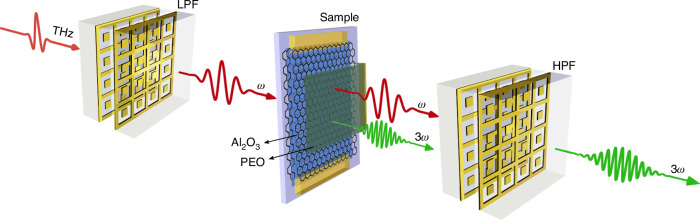
Schematic of the experimental configuration at the sample position to generate and detect THz third harmonic generation. A LPF transmits a multicycle fundamental pulse at ω, which produces a signal at 3ω after nonlinear interactions in a graphene-based sample. The LPF and HPF consist of a superposition of four metasurfaces, but two only are schematically shown here for clarity. The graphene sheet is attached to electrodes (top and bottom), functioning as source and drain terminals for electrical characterization. Gating is facilitated by a polymer electrolyte [LiClO_4_-Polyethylene oxide (PEO)] layer deposited on top of the graphene sample

The experimental configuration is shown schematically in Fig. 1. Sensitive detection of the THG signal requires the use of a lowpass filter (LPF), reducing the spectral width of the pump pulse and eliminating any residual pump at the third harmonic frequency. After the generation of the THG inside the graphene-based sample, a highpass filter (HPF) is used to limit the incident pump reaching the detection scheme. Although the HPF used in our experiment does not completely eliminate the pump, it still reduces the pump-induced noise at the third harmonic frequency to a value close to the noise floor. This technique relying on a HPF significantly increases the sensitivity of time-resolved detection to monitor the creation of new spectral components^24,29^. The general design of these filters are described in previous work^31^ and their spectral transmission properties are shown in Section 2 of the Supplementary Information. In brief, the LPF transmits multicycle THz pump pulse centered at the frequency, ω = 0.8 THz, and strongly attenuates (∼50 dB) spectral components beyond 1.5 THz. The HPF attenuates the pump pulse by >30 dB while transmitting the third harmonic at 3ω.

**Fig. 2:**
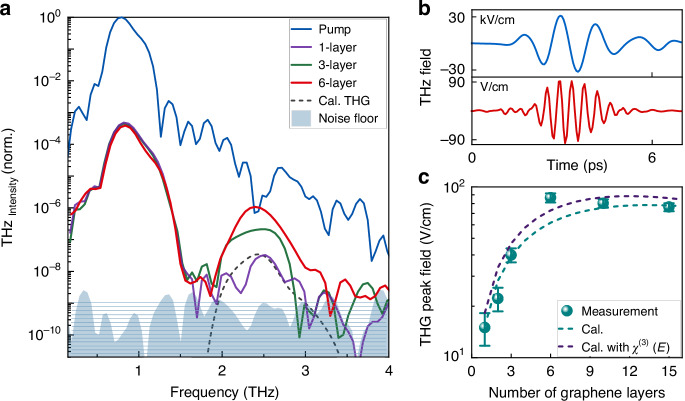
Measured THG in stacked graphene layers. **a** The blue line represents the spectrum of normalized THz pump intensity transmitted through a LPF. A HPF is used to collect the other measurements. THG signature is observed around 2.4 THz for stacked graphene layers: 1-, 3-, and 6-layers. The grey shaded area shows the noise floor. The dashed black line is a calculation of the THG spectrum produced by a single-layer graphene also accounting for the experimental detection efficiency. **b** Time-domain THz transient of the pump pulse (blue line) and third harmonic pulse (red line) collected with 6-layer graphene. The y-axis units for the fundamental pulse are kV/cm and they are V/cm for the third harmonic, **c** THG peak field measured in samples with different numbers of graphene layers (green circles) compared with calculations (dashed lines). The error bars correspond to the standard deviation calculated from five measurements on different spots

We investigate six nonlinear samples consisting of stacked graphene sheets, from 1 to 15 layers, allowing us to vary the nonlinear interaction length. Figure 2a shows the spectrum of the THz pulse (blue line) transmitted through the LPF and used as the pump at the fundamental frequency ω. This graph only contains spectral measurements collected with the 1-, 3-, and 6-layer graphene samples (purple, green and red lines, respectively) for enhanced clarity of the display. A distinct THG spectral peak at 3ω = 2.4 THz can be observed with an intensity increasing roughly quadratically with the number of graphene layers (see Sections 1 and 3 in the Supplementary Information). The HPF, which role is to decrease the residual pump, allows the noise level at frequencies beyond 1.5 THz to settle close to the noise floor (grey shaded area), enabling sensitive monitoring of the nonlinear effects. Figure 2b shows the time-resolved multicycle pump pulse (blue line) with a peak amplitude of 32 kV/cm. This signal is compared to the THG waveform (red line) generated by a 6-layer graphene sample. The time-resolved pump pulse is measured directly by electro-optic sampling in the experiment. The third harmonic waveform, weak in amplitude in comparison to the residual pump, is extracted by numerically applying a spectral bandpass filter around 2.4 THz and then using the inverse Fourier transform. Figure 2c shows the third harmonic peak amplitude obtained with all the samples investigated in this experiment. The largest nonlinear signal is obtained with the stack of 6 layers. This sample yields a THG peak amplitude that is a factor of 5.8 (or 33 in peak intensity) larger than the one obtained with the single graphene sheet. We do not observe further increase of the THG peak amplitude using a stack of 10 or 15 layers due to linear losses experienced by the pump and the third harmonic signal. To monitor the level of inhomogeneity on each graphene-stack samples, data is collected on 5 different spatial spots separated by 700 µm. We find standard variations of the THG signal of 22% for the single graphene layer (error bars in Fig. 2c). Spatial fluctuations in nonlinear properties can be attributed to an inhomogeneous distribution of impurities, such as PMMA residues, and uneven sample growth quality leading to changes in the doping level and electronic transport properties^32^. Much smaller fluctuations of ∼6% are measured with the 10- and 15-layer samples due to a smoothing effect of the spatial inhomogeneities across the different graphene layers. The displayed amplitudes in Fig. 2b, c correspond to the THG field after the graphene sample as we multiply the measured amplitude by 1.9 to account for the total experimental losses at 2.4 THz. Three effects leading to this signal attenuation are considered: (i) a 25% (factor of 1.33) decrease of the THG signal is due to the gating pulse duration (108 fs FWHM), which reduces the detection sensitivity towards higher THz frequencies^33^; (ii) a 11% (factor of 1.12) lower detection efficiency at 2.4 THz is due to phase matching conditions in the THz detection crystal (see Section 4 in the Supplementary Information); (iii) a 21% (factor of 1.27) is lost because of the transmission properties of the HPF (see Section 2 in the Supplementary Information). Taking these factors into account, we found a peak field conversion efficiency of ∼0.2% in 6-layer graphene. When considering the spectrally integrated third harmonic amplitude within the 1/e peak linewidth, the conversion efficiency reaches 0.24%. This value is twice the maximum efficiency previously reported for a single-layer graphene^24^, which was obtained with an accelerator-based super-radiant source producing longer multicycle pulses at a lower fundamental frequency of 0.3 THz. Note that a driving THz field with a larger number of cycles would likely lead to larger optical nonlinearities induced by deposited heat^28^. Additionally, a lower driving frequency is expected to produce a stronger nonlinear THz conduction in graphene^23,26^.

We perform theoretical calculations of the THG spectrum and peak amplitude based on the nonlinear wave equations^8^ using the experimentally measured pump spectrum and peak driving field. Figure 2a shows the calculated spectrum generated within a single graphene sheet (black dashed line) using a nonlinear coefficient ${\chi }^{\left(3\right)}$ = 2.4 × 10^-10^ m^2^/V^2^, which is obtained by fitting the THG peak value. To allow direct comparison between this calculation and raw experimental spectral curves in Fig. 2a, we divide the theoretical result by 3.6 (square of the factor 1.9 described above for the field amplitude) to account for experimental losses. The third-order nonlinear coefficient used in this calculation is 20% lower than the one reported in previous work (${\chi }^{\left(3\right)}$ = 3 × 10^-10^ m^2^/V^2^ at 32 kV/cm) in a similar experiment^29^. This can be in part attributed to the duration of our THz driving field, which is shorter than the one used in most previous work on THz HHG. Since the nonlinear response is expected to originate from a ∼1 ps energy relaxation time^23^, a longer-lasting multicycle driving signal may result in slightly larger nonlinearities^28^. In Fig. 2c, nonlinear calculations using the parameters found for a single-sheet graphene are performed for multilayered graphene samples from which we can extract the THG peak amplitude (see Section 3 in the Supplementary Information). We consider a 4% amplitude loss (8% power loss) for both the THz pump and third harmonic as they pass each graphene layer (see Section 1 in the Supplementary Information). This loss in the THz region is consistent with values reported by previous work^26,32,34^. Furthermore, we also perform the same calculation with a third-order nonlinear coefficient that is a function of the field strength according to previous experimental observation in graphene^29^ (see Section 1 in the Supplementary Information). We find an overall agreement between the model and experimental results, and no major differences when field-dependent nonlinearities of graphene are introduced in the model. The main discrepancies between the experiment and calculations, which arise when the number of layers exceeds 3, can be in part attributed to variations of the nonlinear and electrical transport properties across different graphene sheets, as previously observed in other experiments involving graphene^35^. Our calculations indicate that the maximum THG signal is expected for a 9-layer device, which is 14% larger than the signal calculated for a 6-layer device. As a result, the higher nonlinear conversion efficiency experimentally observed with a 6-layer graphene sample can be attributed to sheet-to-sheet fluctuations of the nonlinear properties of graphene.

### Electrical gating tunability of stacked graphene

**Fig. 3:**
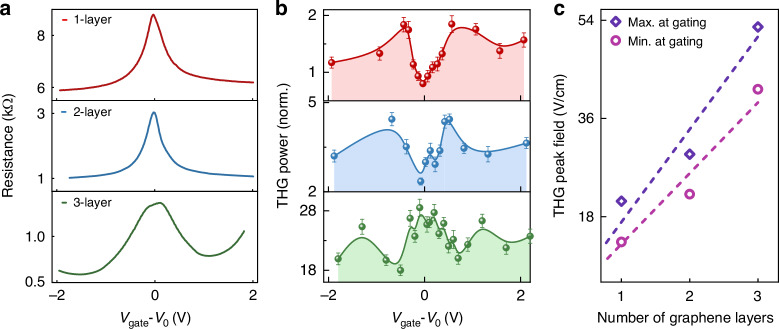
Electrical tunability of terahertz nonlinearity in gated multilayer graphene sheets. **a** Experimentally measured sheet resistance and **b** extracted THG power for 1-layer (red), 2-layer (blue), and 3-layer (green) graphene sheets as we vary the gate voltage (V_gate_). V_0_ represents the applied voltage necessary to reach the maximum resistance of graphene, corresponding to the lowest carrier density. The circles represent experimental data points with error bars representing the standard deviation of the measurements, and the lines depict a b-spline interpolation used as a guide to the eye. **c** Maximum and minimum THG peak field amplitudes measured on all gated multilayered samples while varying the gate voltage. The dashed line shows a linear fit

Graphene’s electronic THz nonlinearity has been attributed to the interaction of the THz field with free carriers and the subsequent interplay among electronic, phononic, and thermodynamic processes^6,23,36^. Therefore, a variation of the nonlinear response can be induced by an electrical gate changing the carrier density^28^. We use 1-, 2-, and 3-layer graphene samples electrically connected to a gate to simultaneously study the effect of carrier density and a multilayered design on nonlinear effects. The density is controlled using a polymer electrolyte (LiClO_4_-Polyethylene oxide (PEO)) gate^37,38^ connected to each graphene layer of the stack (see Methods). The electrical resistance of the samples is monitored by measuring the source-drain current as a function of the gate voltage. From these measurements, one can define the voltage offset V_0_ required to reach the minimum free carrier density, also referred to as the charge neutrality point. As shown in Fig. 3a, the total resistance decreases as we increase the number of layers because they are connected in parallel (see Fig. S5 of the Supplementary Information). By scanning the gate voltage V_gate_, we obtain a standard resistance response with a peak corresponding to the minimum doping concentration while the n- and p- doped region correspond to the negative and positive region of V_gate_-V_0_, respectively. We vary the gate voltage and monitor the THG power calculated by integrating the spectrum centered at 2.4 THz over the 1/e^2^ bandwidth. Figure 3b shows experimental measured THG power (circles) as a function of V_gate_-V_0_ where error bars represent the standard deviation of the measurements. With a single graphene layer, we find a minimum THG power at the charge neutrality point because less carriers are contributing to the material nonlinearities^28^. We find two maxima symmetrically on each side of this point, at |V_gate_-V_0_ | = 0.7 V. The ratio between the two extreme values is ε_1G_ = 2.3, which confirms that controlling the carrier density is essential for optimizing the THG efficiency. As we further increase both the n- and p-doping concentration, the nonlinear signal decreases by about 50%. This behavior agrees with previous reports on the emergence of a metallic phase at high doping concentration gradually reducing the material’s thermodynamic nonlinearity^28^.

Interestingly, the dependence of the THG power as a function of the gate voltage is different when we add more graphene layers. In the case of a 2-layer graphene sample, the dependence on V_gate_ closely resembles the one observed with a single layer. However, we find ε_2G_ = 1.9, which is lower than the same ratio measured with a single layer ε_1G_. For 3 layers, the signal as a function of V_gate_ is more erratic, with a peak, instead of a dip, around V_gate_-V_0_ = 0, and what appears to be random oscillations as a function of V_gate_. Nonetheless, we measure ε_3G_ = 1.6, indicating the significant effect the gate still has on the carrier density to enhance the sample’s nonlinear effects. The lower values of ε as the number of graphene layer is increased can be attributed to a different intrinsic doping concentrations across the layers, resulting in different values for V_0_^35^. As a result, it is unlikely that the neutrality point of multiple superimposed graphene layers can be reached simultaneously with a single gate. Also, because of the device geometry, the change in carrier density may be different across the layers because of a screening effect, potentially causing larger variations inside the layers closer to the gate on top. Therefore, a multilayered architecture able to independently control carrier density in each graphene sheets would be optimal to reach maximum nonlinear effects. In this experiment, the 3-layer sample displays the highest nonlinear conversion efficiency due to its longer interaction length even though gate-induced enhancement of nonlinear effects is slightly lower as discussed above. Figure 3c shows the minimum and maximum THG power obtained while changing V_gate_ with the 1-, 2-, and 3-layer gated graphene samples. These two sets of data when expressed in their corresponding peak field values follow a linear dependence (dashed lines) with the number of layers. The significant separation between these lines attest to the importance in controlling graphene’s carrier density to optimize nonlinear effects.

### Metamaterial-graphene architectures

**Fig. 4:**
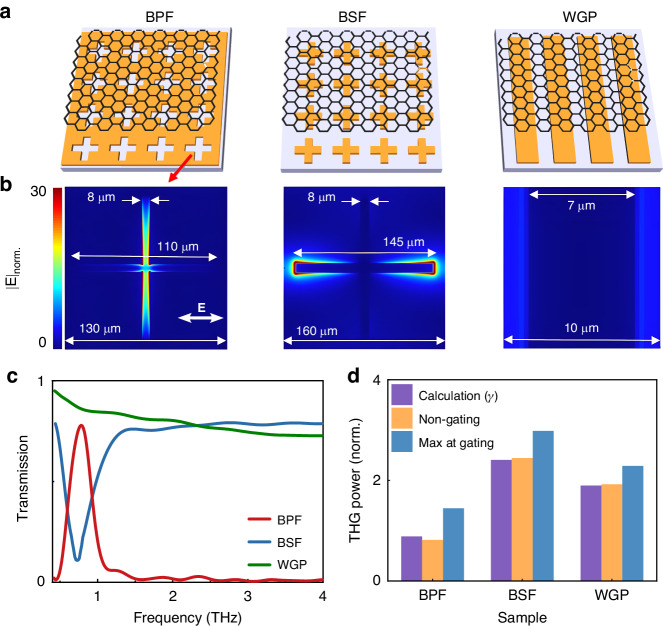
Metamaterial-graphene-based architectures. **a** Schematic of a cross-slot bandpass filter (BPF), cross-shaped bandstop filter (BSF), and a wire-grid polarizer (WGP) with multilayer graphene deposited on their top surface. **b** FDTD simulated electric field distribution inside graphene for the three architectures. Metallic elements are shown in dark blue. **c** Measured THz power transmission of the metamaterial structures displaying fundamental resonances around 0.8 THz for the BPF and BSF. **d** Metasurface-induced enhancement of the THG signal. We show numerical calculations $\gamma$ compared to experimental THG power normalized to the value obtained with a bare 2-layer graphene. We distinguish measurements collected when no gate voltage is applied (V_gate_ = 0) and when V_gate_ is optimized to obtain a maximum THG

In a third series of experiments, we explore the effect of metasurfaces on THG in an architecture combining multilayer graphene and an electrical gate. Three different designs of metasurfaces are considered based on their distinctive spectral transmission properties at the THz pump frequency. Figure 4a shows a schematic of these samples. They consist of a cross-slot bandpass filter (BPF), a cross-shaped bandstop filter (BSF), and a wire-grid polarizer (WGP). Two superimposed graphene sheets acting as the nonlinear medium are transferred on each metasurface. A transparent polymer electrolyte gate deposited on top of the structure is used to vary the carrier density (see Section 5 of the Supplementary Information). Figure 4c shows the measured THz transmission spectrum of each device with the fundamental resonance of the BPF and BSF centered at the THz pump frequency of 0.8 THz. We use FDTD simulations (Lumerical) to visualize the electric field distribution within a metasurface’s unit cell at the height corresponding to the 2-layer graphene sample (Fig. 4b). We observe a significant enhancement of the incident THz field in the gap between metallic elements, locally reaching more than an order of magnitude. Since the third harmonic field amplitude ${E}_{3\omega }$ has a cubic dependence on the driving field ${E}_{\omega }$, this effect directly leads to a more efficient THG. Figure 4d shows the measured third harmonic power normalized to the value obtained with the bare 2-layer graphene. We distinguish measurements collected when no gate voltage is applied (V_gate_ = 0) (yellow columns) and when V_gate_ is optimized to reach the maximum THG power (blue columns). The BSF-graphene architecture leads to the largest THG power. We measure an enhancement factor of 2.5 when the sample is non-gated and a factor of 3 when V_gate_ is optimized to the maximum THG power. The WGP-graphene architecture increases the THG power by a factor of 2 for non-gated approach, similar to the results presented in previous work for a similar device at the same peak field^29^. The use of BPF slightly reduces THG if the sample is not gated. Although this structure locally displays a high field enhancement, this feature is partially counteracted by a large fraction of the sample covered by the metallic structure, and therefore not contributing to nonlinear effects. For both BPF and WGO, the effect of gating can increase the THG signal by 20% and 75%, respectively.

To theoretically estimate the impact of the metasurface substrate on the nonlinear conversion efficiency, we use numerical simulations to calculate the field distribution and we then integrate ${E}_{\omega }^{3}$ within a metasurface’s unit cell (uc). This value is compared to a reference obtained without the metasurface. In our calculations, we incorporated the induced field dependence of the third-order nonlinear coefficient, ${\chi }^{\left(3\right)}\left({E}_{\omega }\right)$, using a rational fitted model derived from previously work^29^ (see Section 1 in the Supplementary Information for simulation details and fitting model). The ratio $\gamma ={\oint }_{{uc}}\,{{\chi }^{\left(3\right)}{E}}_{\omega ,{MS}}^{3}{da}/{\oint }_{{uc}}\,{{\chi }^{\left(3\right)}{E}}_{\omega ,0}^{3}{da}$ indicates the THG enhancement factor induced by the metasurfaces. In Fig. 4d, we compare $\gamma$ (magenta columns) to the corresponding experimental results. We find a good agreement between the calculated and measured THG power. The slight discrepancies may arise from variations in the enhancement of plasmonic structures between simulations and fabricated samples, particularly at the sharp edges. Additionally, minor shifts in resonance may occur due to deviations in dimensions during the fabrication process.

Numerical simulations reveal that the field amplitude at the driving frequency remains nearly constant along the direction normal to the substrate. Within a 2 µm distance, the amplitude variation remains below 5%. This ensures that similar metasurface-induced enhancement factors of the THG signal will be observed regardless of the number of decoupled graphene layers in the sample. This remains true for thick samples since, after 15 graphene layers, additional layers contribute less to the THG signal due to the absorption of the driving field. Therefore, considering a sample with 6 to 9 decoupled graphene sheets, electrically connected to allow independent control of carrier concentration in each layer, and deposited on a BSF metasurface substrate, we can envision a total THG enhancement factor exceeding 100.

## Discussion

**Fig. 5:**
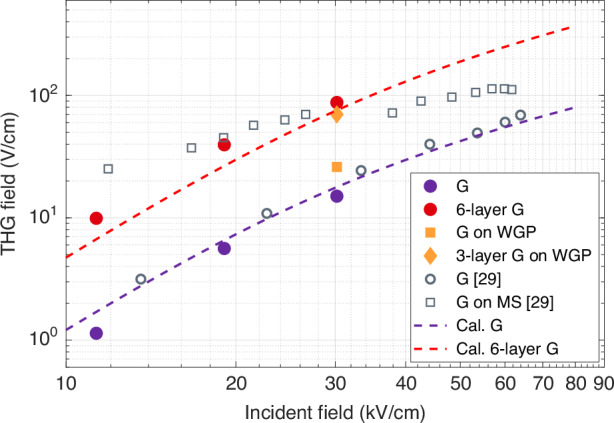
Efficiency of THG strength in graphene over incident driving peak-field. Solid circles indicate the measured THG for 1-layer and 6-layer graphene. Orange squares and diamonds represent the THG enhancement for 1-layer and 3-layer graphene on a wire-grid polarizer (WGP) metamaterial. The gray circles and squares correspond to the THG measurements for 1-layer graphene on grating metasurface (MS) structures, as reported by Deinert et al.^29^. Dashed lines depict the THG considering the field-dependent third-order nonlinear coefficient (${\chi }^{\left(3\right)}\left(E\right)$) for 1-layer and 6-layer graphene samples

Figure 5 summarizes the measured and calculated THG in single and multilayer graphene samples as a function of the incident THz pump peak field. Using a single graphene sheet (purple circles) and a 6-layer graphene (red circles) device, we monitor the THG at several driving fields while moving the position of the sample at the THz focus along the driving beam propagation axis. Using the model described in Section 1 of the Supplementary Information, we also calculate the corresponding THG field (dashed lines). We compare our findings with those reported by Deinert et al.^29^ on THz THG in a single graphene sheet (open circles), which is consistent with our results. When graphene is deposited on a grating metasurface (MS) structure (open squares), similar to a wire-grid polarizer (WGP) design, Deinert et al. observe a ten-fold increase in THG amplitude. Using a similar device architecture, we observe a THG signal (orange squares) that is not as high, which might be due to sample-to-sample fluctuations in electric transport properties in graphene. Similar THG field amplitudes can nonetheless be reached using a 6-layer graphene sample (red circles) or by depositing a three-layer graphene sample on a WGP (orange diamonds). Note that saturation effects in graphene at high fields limit the maximum THG signal when the THz driving field exceeds 20 kV/cm. A multilayer sample can be used to achieve a large THG signal without relying on local field enhancement, which mitigates these saturation effects. Furthermore, in such a multilayer design, each graphene layer partially absorbs the driving field, which also contributes in reducing saturation effects. As a result, a design relying on multiple-layer graphene offers the best perspective for nonlinear frequency conversion at high driving fields, while a graphene mounted on a metasurface offers the largest enhancement factor at low driving fields, away from the saturation regime. Interestingly, calculations show that a bandstop or bandpass metasurface substrate can lead to an enhancement of the THG amplitude generated in graphene by more than two orders of magnitude at a weak incident field <1 kV/cm (see Section 6 of the Supplementary Information).

We investigate third harmonic generation (THG) in graphene in the THz range while relying on three types of device architectures to make this nonlinear process more efficient. We experimentally investigate nonlinear samples with (i) a different number of graphene layers to increase the nonlinear interaction length, (ii) an electrical bias with a gate voltage to optimize the carrier density, and (iii) metallic metasurface substrates to locally enhance the pump field. Especially, we explore the potential to combine these techniques together within one structure. The largest improvement of the THG signal is observed by increasing the number of graphene layers. In particular, the most efficient THG is measured with a 6-layered graphene sample, representing a trade-off between the nonlinear interaction length and linear absorption, which leads to a third harmonic power 33 times higher than obtained with a single graphene layer. We then use a simple configuration allowing a transparent electrical gate to tune nonlinear properties of graphene-based samples. In a gated single graphene layer, THG can increase by a factor of 2.3. Although this factor is lower when the sample contains multiple graphene layers, the gating voltage always leads to a significant increase of the THG signal, larger than 60% in our experiment. Plus, a design allowing independent tuning of the free carrier concentration in each layers could be used to ensure a two-fold THG enhancement in multilayered designs. In a third series of experiments, different types of metasurfaces are used as substrate to explore the effect of local field enhancement on THG in graphene. Interestingly, the most efficient design is a bandstop filter (BSF) at the fundamental frequency, featuring a 3-fold increase in THG power. We propose a model based on numerical calculations to estimate the nonlinear signal enhancement induced by a metasurface, which provides good agreement with experimental results. More importantly, our work demonstrates the possibility to combine these strategies to potentially enhance THG by more than two orders of magnitude. The sample architectures can also apply to other types of 2D nonlinear materials, such as transition-metal dichalcogenides^39^, and they can be combined with other techniques to enhance nonlinear effects, such as the use of topological insulating substrates^14,40^. Multilayered samples, potentially combining different materials, may also be an interesting route leading to enhanced THG, especially at high fluences. Additionally, given that the efficiency of THz generation and detection in table-top systems can still be improved, there remains many nonlinear phenomena leading to THz frequency conversion that are yet to be explored in greater detail. Altogether, such advancements are essential to enable efficient chip-integrated nonlinear THz signal converters that will be used in future communication technologies.

## Materials and methods

### Sample preparation

We use commercial monolayer CVD-grown graphene on copper foil, coated with a poly(methyl methacrylate) (PMMA) layer, obtained from Graphenea. Graphene layers of 1 cm × 1 cm dimensions are wet-transferred onto a 188 µm thick Zeonor substrate^32^ (a cyclo-olefin copolymer transparent to THz radiation with a refractive index of n ∼ 1.53 at 1 THz). This is followed by the deposition of a 60 nm-thick Al_2_O_3_ layer using an electron-beam evaporator (NexdepSeries, Angstrom). To form multilayer graphene samples, additional graphene layers and alternating Al_2_O_3_ layers are stacked on the same substrate.

The gating voltage is applied with a 5 nm Ti/20 nm Pd/150 nm Au electrodes deposited on the sides of the graphene layers using a single evaporation system and a shadow mask placed atop the graphene surface. The source and drain contacts make direct contact with the graphene layer, while the gating contacts has no direct contact. A spray-coating technique is employed to apply a 400 nm-thick transparent polymer electrolyte onto the graphene layers. The electrolyte is made of Polyethylene oxide (PEO) and LiClO_4_ in an 8:1 weight ratio, which is dissolved in a methanol solution. Subsequently, the sample is affixed onto a custom-printed circuit board (PCB) with a central hole to perform optical transmission measurements. The electrical measurements are performed at constant voltage while monitoring the current with Keithley source meters (models 2400).

The metasurfaces are fabricated on a 188 µm thick Zeonor substrate using a conventional positive photolithography process. Aluminum is deposited onto the patterned substrate using a sputtering technique, followed by a lift-off process to achieve 200 nm-thick metallic arrays. Subsequently, a 60 nm-thick Al_2_O_3_ layer was deposited on the metasurfaces using an electron-beam evaporator, after which graphene sheets were wet-transferred onto them.

### Experiment

In our experimental setup, high-field (360 kV/cm) single-cycle THz pulses are generated using the tilted pulse front technique in a LiNbO_3_ crystal. These pulses originated from an amplified 10 kHz Yb-laser system with the following parameters: central wavelength of 1030 nm, a pulse width of 170 fs, and a pulse energy of 1 mJ. Detection involved electro-optical sampling method by utilizing pulses from an optical parametric amplifier (OPA) at 960 nm wavelength, with a pulse duration of 108 fs (FWHM), and employing a 1 mm-thick GaP nonlinear detection crystal. The gating pulses at 960 nm satisfy phase matching condition in GaP to achieve high detection sensitivity at the third harmonic frequencies at 2.4 THz. All experiments are conducted at room temperature inside a dry-air purged enclosure.

### Simulations

Numerical simulations are performed with a 3D finite-difference time-domain (FDTD) solver (Lumerical Inc.) to design the plasmonic metasurface resonators with fundamental resonances around 0.8 THz and explore the electric field distribution of the THz pump pulse on the structures. We utilize periodic boundary conditions along the in-plane axis. Additionally, a perfectly matched layer (PML) is applied in the direction of optical propagation to absorb all incident fields, effectively preventing any reflections at that interface. For the simulation of electric field spatial distributions, we employed a minimum override mesh size of 50 nm along the surface of the structures and 4 nm along the THz propagation direction.

## Supplementary information

Supplementary information

## Data Availability

The experimental data that support the findings of this work is available upon reasonable request from the corresponding authors.

## References

[1] Pizzuto, A., Ma, P. C. & Mittleman, D. M. Near-field terahertz nonlinear optics with blue light. *Light Sci. Appl.* **12**, 96 (2023). 37072386 10.1038/s41377-023-01137-y PMC10113216

[2] Chai, X. et al. Subcycle terahertz nonlinear optics. *Phys. Rev. Lett.* **121**, 143901 (2018). 30339430 10.1103/PhysRevLett.121.143901

[3] Heindl, M. B. et al. Ultrafast imaging of terahertz electric waveforms using quantum dots. *Light Sci. Appl.* **11**, 5 (2022). 34974517 10.1038/s41377-021-00693-5 PMC8720308

[4] Nicoletti, D. & Cavalleri, A. Nonlinear light–matter interaction at terahertz frequencies. *Adv. Opt. Photonics* **8**, 401–464 (2016).

[5] Koshihara, S. et al. Challenges for developing photo-induced phase transition (PIPT) systems: From classical (incoherent) to quantum (coherent) control of PIPT dynamics. *Phys. Rep.* **942**, 1–61 (2022).

[6] Pogna, E. A. A. et al. Hot-carrier cooling in high-quality graphene is intrinsically limited by optical phonons. *ACS Nano* **15**, 11285–11295 (2021). 34139125 10.1021/acsnano.0c10864 PMC8320233

[7] Ganichev, S. D. & Prettl, W. Terahertz nonlinear optics. in Intense Terahertz Excitation of Semiconductors (eds Ganichev, S. D. & Prettl, W.) (Oxford: Oxford University Press, 2005), 269–290, 10.1093/acprof:oso/9780198528302.003.0007.

[8] Boyd, R. W. *Nonlinear Optics*. 4th edn. (London: Academic Press, 2020), 10.1016/C2015-0-05510-1.

[9] Yang, X. et al. Lightwave-driven gapless superconductivity and forbidden quantum beats by terahertz symmetry breaking. *Nat. Photonics* **13**, 707–713 (2019).

[10] Vaswani, C. et al. Terahertz second-harmonic generation from lightwave acceleration of symmetry-breaking nonlinear supercurrents. *Phys. Rev. Lett.* **124**, 207003 (2020). 32501057 10.1103/PhysRevLett.124.207003

[11] Reinhoffer, C. et al. Strong terahertz third-harmonic generation by kinetic heavy quasiparticles in CaRuO_3_. *Phys. Rev. Lett.* **132**, 196501 (2024). 38804953 10.1103/PhysRevLett.132.196501

[12] Meng, F. Q. et al. Higher-harmonic generation in boron-doped silicon from band carriers and bound-dopant photoionization. *Phys. Rev. Res.* **5**, 043141 (2023).

[13] Murakami, Y., Nagai, K. & Koga, A. Efficient control of high harmonic terahertz generation in carbon nanotubes using the Aharonov-Bohm effect. *Phys. Rev. B* **108**, L241202 (2023).

[14] Tielrooij, K. J. et al. Milliwatt terahertz harmonic generation from topological insulator metamaterials. *Light Sci. Appl.* **11**, 315 (2022). 36316317 10.1038/s41377-022-01008-y PMC9622918

[15] Giorgianni, F. et al. Strong nonlinear terahertz response induced by Dirac surface states in Bi_2_Se_3_ topological insulator. *Nat. Commun.* **7**, 11421 (2016). 27113395 10.1038/ncomms11421 PMC4853424

[16] Kovalev, S. et al. Terahertz signatures of ultrafast Dirac fermion relaxation at the surface of topological insulators. *npj Quantum Mater.* **6**, 84 (2021).

[17] Kovalev, S. et al. Non-perturbative terahertz high-harmonic generation in the three-dimensional Dirac semimetal Cd_3_As_2_. *Nat. Commun.* **11**, 2451 (2020). 32415119 10.1038/s41467-020-16133-8 PMC7229177

[18] Zhou, R. et al. Engineering the harmonic generation in graphene. *Mater. Today Phys.* **23**, 100649 (2022).

[19] Yoshikawa, N., Tamaya, T. & Tanaka, K. High-harmonic generation in graphene enhanced by elliptically polarized light excitation. *Science* **356**, 736–738 (2017). 28522530 10.1126/science.aam8861

[20] Cha, S. et al. Gate-tunable quantum pathways of high harmonic generation in graphene. *Nat. Commun.* **13**, 6630 (2022). 36333325 10.1038/s41467-022-34337-y PMC9636431

[21] Calafell, I. A. et al. High-harmonic generation enhancement with graphene heterostructures. *Adv. Opt. Mater.* **10**, 2200715 (2022).

[22] Soavi, G. et al. Hot electrons modulation of third-harmonic generation in graphene. *ACS Photonics* **6**, 2841–2849 (2019).

[23] Hafez, H. A. et al. Terahertz nonlinear optics of graphene: from saturable absorption to high-harmonics generation. *Adv. Opt. Mater.* **8**, 1900771 (2020).

[24] Hafez, H. A. et al. Extremely efficient terahertz high-harmonic generation in graphene by hot Dirac fermions. *Nature* **561**, 507–511 (2018). 30202091 10.1038/s41586-018-0508-1

[25] Di Gaspare, A. et al. Electrically tunable nonlinearity at 3.2 terahertz in single-layer graphene. *ACS Photonics* **10**, 3171–3180 (2023). 37743945 10.1021/acsphotonics.3c00543 PMC10515698

[26] Mics, Z. et al. Thermodynamic picture of ultrafast charge transport in graphene. *Nat. Commun.* **6**, 7655 (2015). 26179498 10.1038/ncomms8655 PMC4518297

[27] Mao, W. W., Rubio, A. & Sato, S. A. Terahertz-induced high-order harmonic generation and nonlinear charge transport in graphene. *Phys. Rev. B* **106**, 024313 (2022).

[28] Kovalev, S. et al. Electrical tunability of terahertz nonlinearity in graphene. *Sci. Adv.* **7**, eabf9809 (2021). 33827824 10.1126/sciadv.abf9809 PMC8026126

[29] Deinert, J. C. et al. Grating-graphene metamaterial as a platform for terahertz nonlinear photonics. *ACS Nano* **15**, 1145–1154 (2021). 33306364 10.1021/acsnano.0c08106 PMC7844822

[30] Hebling, J. et al. Velocity matching by pulse front tilting for large-area THz-pulse generation. *Opt. Express* **10**, 1161–1166 (2002). 19451975 10.1364/oe.10.001161

[31] Maleki, A. et al. Metamaterial-based octave-wide terahertz bandpass filters. *Photonics Res.* **11**, 526–532 (2023).

[32] Scarfe, S. et al. Systematic THz study of the substrate effect in limiting the mobility of graphene. *Sci. Rep.* **11**, 8729 (2021). 33888755 10.1038/s41598-021-87894-5 PMC8062515

[33] Gallot, G. & Grischkowsky, D. Electro-optic detection of terahertz radiation. *J. Opt. Soc. Am. B* **16**, 1204–1212 (1999).

[34] Hwang, H. Y. et al. Nonlinear THz conductivity dynamics in P-Type CVD-grown graphene. *J. Phys. Chem. B* **117**, 15819–15824 (2013). 24102144 10.1021/jp407548a

[35] Rautela, R. et al. Mechanistic insight into the limiting factors of graphene-based environmental sensors. *ACS Appl. Mater. Interfaces* **12**, 39764–39771 (2020). 32658444 10.1021/acsami.0c09051

[36] Jensen, S. A. et al. Competing ultrafast energy relaxation pathways in photoexcited graphene. *Nano Lett.* **14**, 5839–5845 (2014). 25247639 10.1021/nl502740g

[37] Das, A. et al. Monitoring dopants by Raman scattering in an electrochemically top-gated graphene transistor. *Nat. Nanotechnol.* **3**, 210–215 (2008). 18654505 10.1038/nnano.2008.67

[38] Li, H. M. et al. Electric double layer dynamics in poly(ethylene oxide) LiClO_4_ on graphene transistors. *J. Phys. Chem. C.* **121**, 16996–17004 (2017).

[39] Wen, X. L., Gong, Z. B. & Li, D. H. Nonlinear optics of two-dimensional transition metal dichalcogenides. *InfoMat* **1**, 317–337 (2019).

[40] Shi, J. J. et al. Giant room-temperature nonlinearities in a monolayer Janus topological semiconductor. *Nat. Commun.* **14**, 4953 (2023). 37587120 10.1038/s41467-023-40373-z PMC10432555

