# Supplementary material for: Strategies to enhance THz harmonic generation combining multilayered, gated, and metamaterial-based architectures
Source: Light Sci Appl. 2025 Jan 9;14:44. doi: 10.1038/s41377-024-01657-1 (PMC11718264; doi:10.1038/s41377-024-01657-1)
Supplement: Supplementary file 1 — Supplementary information [file 41377_2024_1657_MOESM1_ESM.pdf]

Supplementary information for

Strategies to enhance THz harmonic generation combining multilayered, gated, and metamaterial-based architectures

Ali Maleki,[1] Moritz B. Heindl,[2] Yongbao Xin,[3] Robert W. Boyd,[1,4,5] Georg Herink,[2] Jean-Michel Ménard[1,4,*]

[1]Department of Physics, University of Ottawa, Ottawa, ON K1N 6N5, Canada
[2]Experimental Physics VIII – Ultrafast Dynamics, University of Bayreuth, Bayreuth, 95447, Germany
[3]Iridian Spectral Technologies Ltd, Ottawa, ON K1G 6R8, Canada
[4]School of Electrical Engineering and Computer Science, University of Ottawa, Ottawa, ON K1N 6N5, Canada
[5]Institute of Optics and Department of Physics and Astronomy, University of Rochester, NY 14627, USA

**Email: Jean-Michel Ménard (jean-michel.menard@uottawa.ca)*

Section S1: Calculations of the third harmonic generation (THG)

Fig. S1a depicts the schematic of a multilayer graphene nonlinear sample, illustrating the interaction of a pump pulse with graphene layers and resulting THG signals on each layer. The THG signals produce in each graphene sheets accumulate as the THG pulse propagates in the structure. In detail, the THz pump pulse (E_ω) first impinges on the Zeonor substrate, experiencing an 8% power transmission loss due to Fresnel reflections at the air-substrate interface. Then, successive third harmonic fields ($E_{3\omega}^{i}$) are produced by the THz driving field (E_{ω}^{i}) incident on each graphene layers, where i = 1, 2, 3, … refers to the position of the graphene sheet in the multiplayer stack. During the THz pulse propagation through a stack of graphene sheets, we consider that 4% of the pump pulse amplitude is absorbed in the first graphene layer. The remaining pump pulse then proceeds to interact with the second layer to generate a slightly weaker THG ($E_{3\omega}^{2}$) and where another 4% gets absorbed. This absorption and signal generation process continues for subsequent graphene layers. It is important to note that we also consider a 4% absorption of the third harmonic field amplitude as this component propagates through each graphene layer. Considering both the absorption of the pump pulse (α_ω) and third harmonic components ($\alpha_{3\omega}$), we establish a relationship for THG in n-layer graphene structures as follows:

$$E_{3\omega}^{Total} = \sum_{i=1}^{n} E_{3\omega}^{i} = \frac{3\omega d}{8cn_{3\omega}}\chi^{(3)} \sum_{i=1}^{n} [(1-\alpha_\omega)^{3(i-1)}(1-\alpha_{3\omega})^{n-i}E_{3\omega}^{1},$$

where c is the speed of light, d = 0.3 nm is the thickness of each graphene layer, $n_{3\omega}$ = 10 is the refractive index of the graphene layer, ω is the pump radial frequency, and $\chi^{(3)}$ is the third order susceptibility. For these calculations, we used the linear properties of graphene reported in previous work[1] and nonlinear coefficient of $\chi^3 = 2.4 \times 10^{-10}$ m^2 V^{-2} obtained from our THG data obtained with a single graphene layer.

Since the coefficient $\chi^{(3)}$ of graphene has been observed to be a function of the driving field amplitude, we extract this dependence from previously reported data[1] (circles in Fig. S1b). We calculate a fitting function: $\chi^{(3)} = \frac{7.2e^{-10}}{1+1e^{-9}E_\omega+12e^{-4}{E_\omega}^2}$ in m^2/V^2, where E_ω is the driving electrical

field amplitude in kV cm^{-1}. This model considers a maximum value for $\chi^{(3)}$ as suggested by the thermodynamic calculations of Ref. [1].

This expression for $\chi^{(3)}$ is not only used to estimate the THG in a multilayer graphene structure (purple line in Fig. 2c), but also determine the effect of an inhomogeneous field distribution induced by a metasurface substrate on the THG (purple column in Fig. 4d). We define the metasurface-induced enhancement factor as:

$$\gamma = \oint_{uc} \chi^{(3)} \left(E_{\omega,MS}\right)^3 \, da / \oint_{uc} \chi^{(3)} (E_\omega)^3 \, da,$$

which uses both the calculated electric field spatial distribution inside a graphene layer above the metasurface ($E_{\omega,MS}$), and a homogeneous field impinging on a graphene layer with no metasurface substrate (E_ω). The field modulation induced by the metasurface is simulated using a mesh size of 50 nm along the surface of the structure and 40 nm along the optical propagation direction. Decreasing the mesh size does not alter the results of the calculations. However, increasing the mesh size by a factor of 2 in the in-plane direction, to reach lower spatial resolution, decreases the calculated factor γ by about 5%.

Fig. S1c shows the transmission of the fundamental frequency through samples with different number of graphene layers, along with corresponding FDTD simulations of transmission, reflection, and absorption. We find a good agreement between the simulation and experimental results. The slight discrepancy can be attributed to intrinsic properties of each graphene layer, such as a varying doping level. A fit based on the Beer-Lambert equation reveals a 4% absorption of the THz field through each graphene layer. Reflection gradually increases for samples containing more than 6 layers of graphene, which is consistent with previous studies[2].

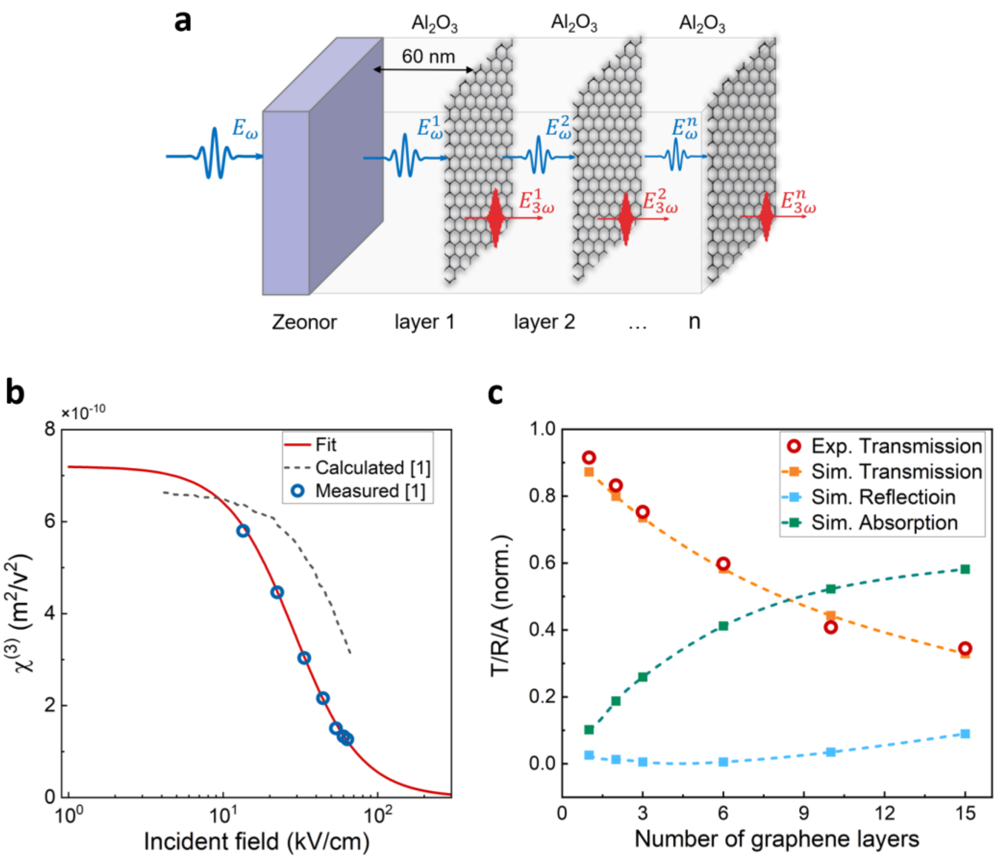

Fig. S1 a Schematic of the THz propagation in multilayer graphene sheets. THz pump pulses on each graphene layer are shown with blue pulses, and THG pulses are shown in red. The schematic of the nonlinear sample comprises a substrate (Zeonor in our experiment) and graphene layers (n-layer), that are separated by Al_2O_3. **b** Measured (circles) and calculated (dashed line) third order nonlinear coefficients ($\chi^{(3)}$) based on the thermodynamic model for a single layer graphene reproduced from J-C. Deinert et al.[1]. Solid line in red shows our fitting function. **c** Measured (red circles) transmission of the stacked graphene samples, obtained from the THz peak intensity at the fundamental frequency (0.8 THz). The solid points represent FDTD simulations of transmission, reflection, and absorption as a function of the number of graphene layers. The dashed lines indicate a spline interpolation to guide the eye.

Section S2: Lowpass filter (LPF) and highpass filter (HPF)

The schematic of the LPF and HPF structures and their corresponding intensity spectral transmission are shown in Fig. S2. The power transmission of the HPF filter is ~60% in the bandpass region, while it attenuates the pump pulse by more than 30 dB in the stopband region. Further improvements of these THz filters would help achieve a more sensitive THG detection.

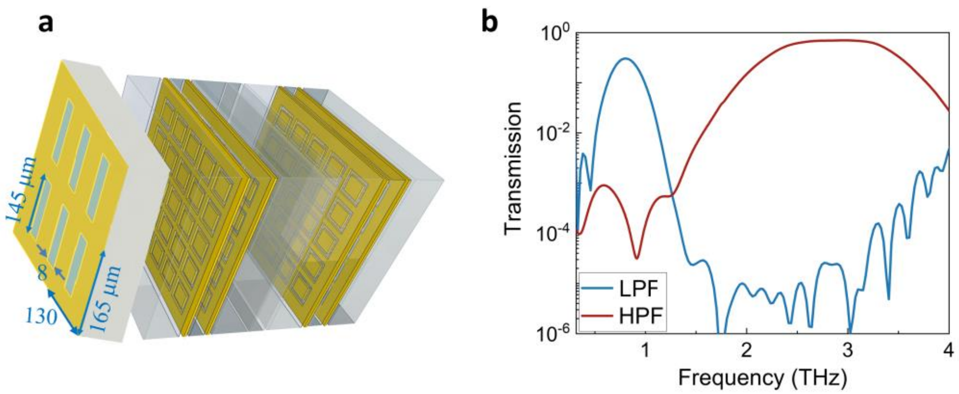

Fig. S2 a Schematic of the spectral filters. The highpass filter (HPF) is made of 4-layer square-slot plasmonic metasurfaces as described in our previous work[3]. During fabrication of the lowpass filter (LPF), we added a bar-slot bandpass filter on top of the 4-layer plasmonic structures to create a sharper bandpass around 0.8 THz and strongly attenuate any residual signal around 2.4 THz. **b** Intensity transmission of the LPF and HPF filters in the THz region.

Section S3: Power vs peak intensity of THG in stacked graphene layers

We calculated the spectral weight by integrating the THG signal in the spectral domain within the e^{-2} bandwidth as a function of the number of graphene layers. The THG peak intensity is obtained by squaring the results presented in Fig. 2c. Overall, the two parameters show the same behavior.

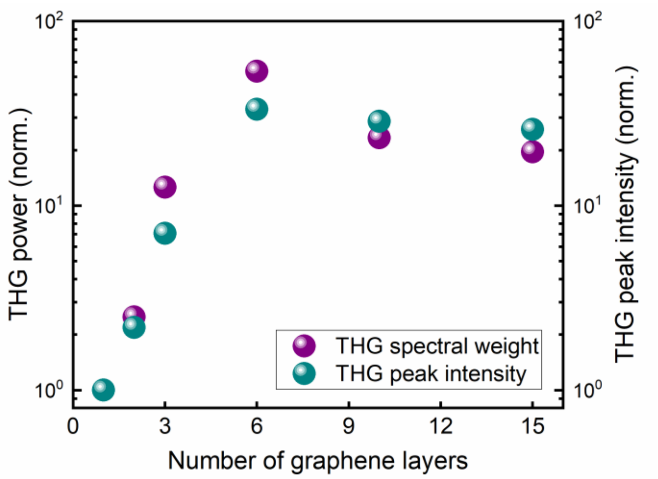

Fig. S3 Third harmonic generation (THG) signal as a function of the number of graphene layers calculated in the frequency domain by integrating the spectral weight to obtain the power (left axis), and in the time-domain by first calculating the field amplitude and then the corresponding peak intensity (right axis).

Section S4: Sensitivity of the GaP detection crystal

We use an electro-optical sampling method to monitor the THz spectrum. The gating pulse, produced by an optical parametric amplifier (OPA), is centered at 960 nm wavelength and has a pulse duration of 108 fs (FWHM) as shown in Fig. S4a. The autocorrelation is obtained by two-photon absorption in a Si photodiode. The deviation from the expected peak-to-background ratio of 8:1 is attributed to a small amount of three-photon-absorption. The wavelength is selected to optimize phase matching conditions at 2.4 THz in our THz detection crystal, which is a 1 mm-thick 110-oriented GaP crystal. The calculated detection efficiency at 2.4 THz is however still 11% lower than the detection efficiency at the pump pulse frequency of 0.8 THz. These calculations use the linear properties of GaP in the THz region[4] are shown in Fig. S4b. The factor of 11% is considered when we compare the field amplitude of the third harmonic and fundamental components.

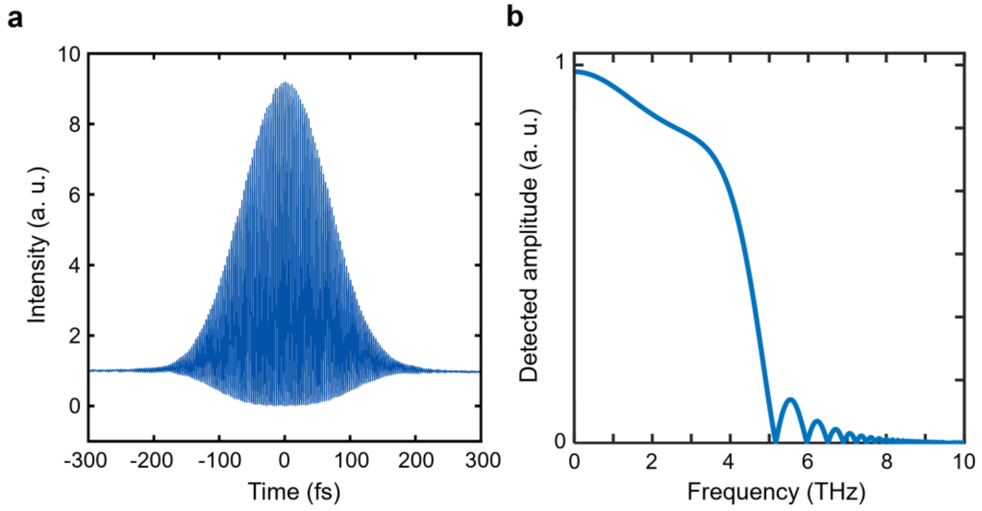

Fig. S4 a Measured interferometric autocorrelation of the gating pulse, representing intensity pulse duration of 108 fs at FWHM. **b** Simulated detection sensitivity of the GaP crystal at the gating 960 nm wavelength.

Section S5: Fabrication of multilayer graphene architectures

We use monolayer CVD graphene on copper foil coated with a poly(methyl methacrylate) (PMMA) layer, and wet transferred them on substrates. This is followed by the deposition of a 60 nm-thick Al_2O_3 layer in between graphene layers. The CVD graphene layer initially exhibited p-type doping, typically originating from chemical residues in the preparation process, wet-transferring processes, interactions with the substrate, and deposited polymer electrolyte[5]. In the next step, a pair of 5 nm Ti/20 nm Pd/150 nm Au electrodes were deposited on the sides of the graphene layers, serving as source and drain terminals for electrical characterization. The graphene sheets were connected in parallel on the sides of the metallic electrodes, as shown schematically in Fig. S5. For each graphene sample, we introduced a gate offset V_0 to effectively compensate for the intrinsically hole-doping effect and shift the Fermi energy towards a neutrally charged Dirac point. This V_0 voltage varied from sample to sample, typically ranging between 0.1 to 0.3 V.

We use the same fabrication process to transfer graphene sheets on plasmonic metasurfaces. 60 nm-thick Al_2O_3 layers serve as spacing between graphene layers and the plasmonic structures to avoid the possibility of any graphene-metal interactions. To apply electrical gating on the graphene-metasurface samples, we employ a spray-coating technique to deposit 400 nm-thick transparent polymer electrolyte (as described in Methods) onto the graphene layers. Finally, in the high-harmonic generation experiment, THz pump light illuminates the structure from the substrate side, initially reaching the plasmonic side, and then interacting with the graphene sheets to achieve THG.

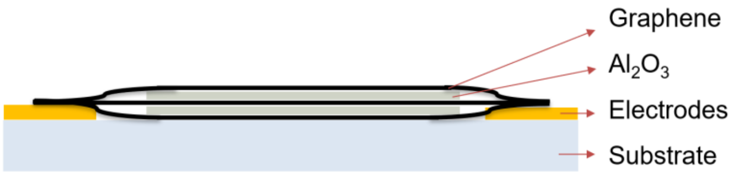

Fig. S5 Schematic of the side-view configuration depicting graphene layers electrically connected in parallel and all attached to metallic electrodes.

Section 6: Metamaterial-based field enhancement

We employ three distinct plasmonic geometries—BSF, BPF, and WGP structures—to locally enhance the THz driving field and improve the nonlinear THG efficiency. Using FDTD simulations, we calculated the electric field distribution within the metasurface unit cell (uc) at 0.8 THz and determined the field-induced THG enhancement ratio in graphene, defined as $\gamma = \oint_{uc} \chi^{(3)}\, E_{\omega,MS}^3\, da / \oint_{uc} \chi^{(3)}\, E_{\omega,0}^3\, da$ (see section Metamaterial-Graphene Architectures). This enhancement ratio for each structure is presented in Fig. S6 as a function of the incident field, demonstrating significant enhancement at lower driving fields but a saturation at higher fields to a value corresponding to the actual fraction of graphene not in direct contact with a metallic sub-structure of the metasurface. Due to the sharper geometries in the BPF and BSF structures, which are more effective at locally intensifying the field, this ratio can increase by up to two orders of magnitude at low incident THz driving field. However, saturation effects limit the THG field enhancement to a value below 5 at driving fields above 10 kV cm^{-1}.

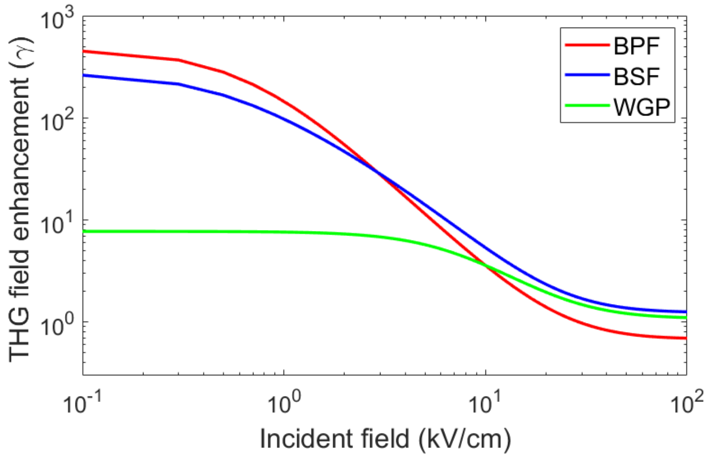

Fig. S6 Calculated THG field enhancement in graphene on different metamaterial structures: bandstop filter (BSF), bandpass filter (BPF), and wire-grid polarizer (WGP), as a function of the driving incident field.

References

1. Deinert, J. C. et al. Grating-graphene metamaterial as a platform for terahertz nonlinear photonics. *ACS Nano* **15**, 1145-1154 (2021).
2. Kim, S.-S., Kim, Y.-K., Park, I.-S. & Shin, S.-C. Optical properties of a thin-film stack illuminated by a focused field. *Journal of the Optical Society of America A* **17**, 1454-1460 (2000).
3. Maleki, A. et al. Metamaterial-based octave-wide terahertz bandpass filters. *Photonics Research* **11**, 526-532 (2023).
4. Gallot, G. & Grischkowsky, D. Electro-optic detection of terahertz radiation. *Journal of the Optical Society of America B* **16**, 1204-1212 (1999).
5. Ryu, S. *et al.* Atmospheric oxygen binding and hole doping in deformed graphene on a SiO2 substrate. *Nano Letters* **10**, 4944–4951 (2010).
